# Evolutionary development of the cephalopod arm armature: a review

**DOI:** 10.1186/s13358-021-00241-z

**Published:** 2021-12-20

**Authors:** Dirk Fuchs, René Hoffmann, Christian Klug

**Affiliations:** 1 Bayerische Staatssammlung für Paläontologie und Geologie, Richard-Wagner-Straße 10, 80333 Munich, Germany; 2 Institute of Geology, Mineralogy, & Geophysics, Ruhr-Universität Bochum, 44801 Bochum, Germany; 3 Paläontologisches Institut und Museum, Universität Zürich, Karl-Schmid-Strasse 4, 8006 Zurich, Switzerland

## Abstract

The cephalopod arm armature is certainly one of the most important morphological innovations responsible for the evolutionary success of the Cephalopoda. New palaeontological discoveries in the recent past afford to review and reassess origin and homology of suckers, sucker rings, hooks, and cirri. Since a priori character state reconstructions are still ambiguous, we suggest and discuss three different evolutionary scenarios. Each of them is based on the following assumptions: (1) Neocoleoidea uniting extant Decabrachia and Octobrachia is monophyletic (= proostracum-bearing coleoids); (2) extinct Belemnitida and Diplobelida are stem decabrachians; (3) proostracum-less coleoids (Hematitida, Donovaniconida, Aulacoceratida) represent stem-neocoleoids; (4) Ammonoidea and Bactritoidea are stem coleoids. We consider a scenario where belemnoid hooks derived from primitive suckers as well-supported. Regarding belemnoid hooks and suckers as homologues implies that belemnoid, oegopsid, and probably ammonoid arm hooks arose through parallel evolution. Our conclusions challenge the widespread opinion, whereupon belemnoid hooks evolved de novo, and instead support earlier ideas formulated by Sigurd von Boletzky.

## Introduction

“From head to foot—and back again” (Boletzky, 2006) is the title of one of Sigurd von Boletzky’s numerous scientific publications that puts one of his most favourite subject in a nutshell: the development of the cephalopod arm crown and its armature (e.g., Boletzky, 1992, 1993a, 1993b, 1999, 2002, 2003, 2006). The complex transformation of the molluscan foot into the cephalopod arm crown was undoubtedly crucial for the evolutionary success of the Cephalopoda. In particular, the sophisticated interplay of both the arms and their suckers is unique. Ideas about the evolution of this tetrapod-like sensory–motor performance are manifold, but hampered by the limited fossil record of cephalopod arm armatures.

Eleven years ago, Fuchs et. al. (2010) reported an arm crown of a belemnoid coleoid from the Late Jurassic Solnhofen Plattenkalks preserving unambiguous suckers and hooks. This record considerably impacted our understanding about the evolution of the coleoid arm armature. It questioned the so-called “Neocoleoidea-concept” (Haas, 1997) whereupon extinct Belemnoidea (= Palaeocoleoidea in other terminologies) represents the sister group of the “Neocoleoidea” (= Dibranchiata in older literature; Jeletzky, 1966; Naef, 1922) that combines the extant taxa Octobrachia (= Octopodiformes or Vampyropoda) and Decabrachia (= Decapodiformes; see also Hoffmann, 2015). Before Fuchs et. al. (2010) published the details of arm morphology, the key characters that distinguish Belemnoidea and Neocoleoidea were referred to the presence of hooks in the former (“Uncinifera”) and suckers in the latter (e.g., Berthold & Engeser, 1987; Engeser & Bandel, 1988; Haas, 1997, 2002; Jeletzky, 1966; Young et al., 1998). Engeser and Clarke (1988) and Haas (1989), who thoroughly reviewed and evaluated the evolutionary history of the coleoid arm armature, considered aulacoceratid, phragmoteuthid, belemnitid, and diplobelid belemnoids as sucker-less and as the extinct sister group of the Neocoleoidea. Earlier evidence of belemnoid suckers (Donovan & Crane, 1992; Mantell, 1852; Pearce, 1847) were either neglected or doubted (e.g., Engeser & Clarke, 1988). With the discoveries by Fuchs et. al. (2010), belemnitid and diplobelid belemnoids became increasingly accepted as stem groups of the Decabrachia (e.g., Fuchs, 2019; Fuchs et al., 2013a; Klug et al., 2016; Kröger et al., 2011). Hence, the Neocoleoidea represents a monophyletic group only when belemnitid and diplobelid belemnoids are included. Such a topology unites all proostracum-bearing coleoids and simultaneously meets an important request from Sigurd von Boletzky, whereupon the differentiation of the decabrachian and octobrachian type of arm crown must have occurred independently at two speciation events (1992, p. 756). Of course, being influenced by his extensive knowledge about the early development of cephalopod structures, Boletzky (1992, 1993a, 1993b, 1999, 2002, 2006) repeatedly doubted a strict sister group relationship between the Octobrachia and Decabrachia, because their different arm modifications (reduction of arm pair II in octobrachians and modification of arm pair IV in decabrachians) must have derived from two different ancestors. “*It thus appears conceivable that a belemnoid morphogenotype provided the basis for the alternative modifications of only one arm pair at two different occasions during coleoid evolution*” (Boletzky, 2002, p. 13). The revived idea of belemnitid and diplobelid belemnoids as sucker-bearing ancestors of decabrachians, new insights about the chemistry of sucker surfaces (e.g., Miserez et al., 2009) as well as discoveries of ammonoid arm hooks (Kruta et al., 2020; Smith et al., 2021) reopen a series of questions that will be addressed here. When did suckers originate? Did belemnoid hooks—contrary to prevailing ideas—derive from suckers? Or are belemnoid, ammonoid, and oegopsid hooks indeed homoplasies or still homologues?

## Suckers

**Fig. 1:**
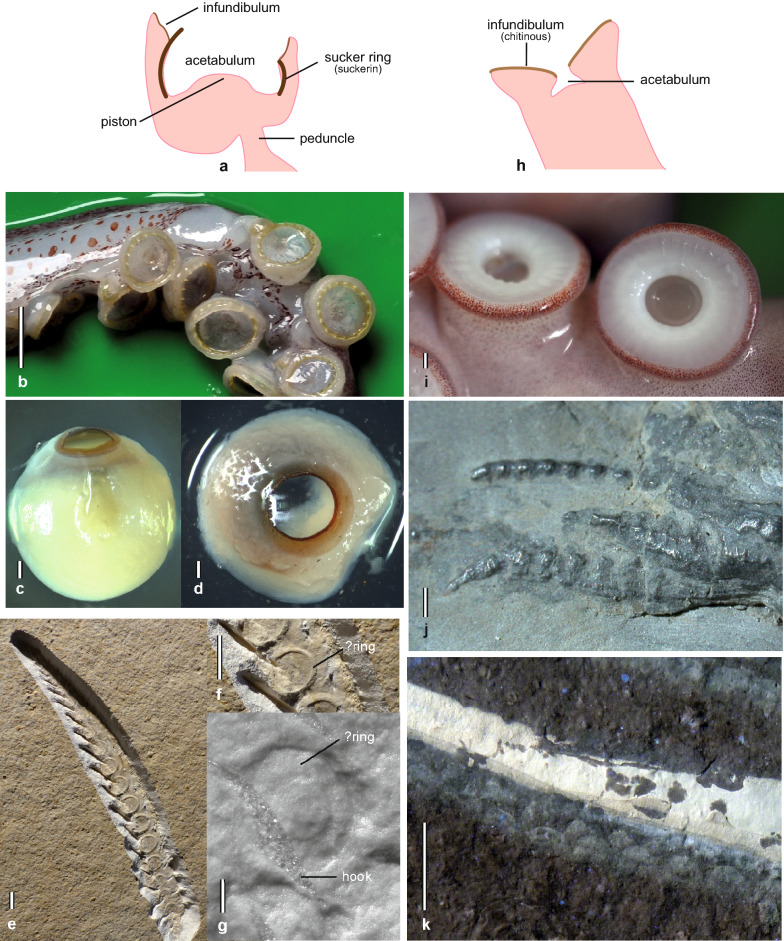
The three main sucker types of coleoid cephalopods. **a**–**d** Decabrachian type of sucker. **e**–**g** Belemnoid type of sucker. **h**–**k** Octobrachian type of sucker. **a** Sketch of the of the decabrachian type of sucker. **b** *Todarodes pacificus* (recent, Ommastrephidae, Oegopsida), suckers (including toothed rings) of the right tentacle. Scale bar: 5 mm. **c**, **d** *Sepiola birostrata* (recent, Sepiolidae, Sepiolida), isolated sucker (including smooth sucker rings) in lateral (**c**) and oral (**d**) view. Scale bar: 1 mm. **e** arm of *Acantoteuthis speciosa* (Belemnoteuthidae, Belemnitida), specimen JMS092, Blumenberg, Solnhofen Plattenkalks, Tithonian. Scale bar: 5 mm. **f** Close up of e showing uniserial ring-like structures. Scale bar: 5 mm. **g** Detailed view of a sucker of *Acantoteuthis speciosa*, specimen of Fuchs et. al. (2010), Eichstätt, Solnhofen Plattenkalks, Tithonian. Scale bar: 1 mm. **h** Sketch of the octobrachian type of sucker. **i** *Octopus conispadiceus* (Recent, Octopodidae, Incirrata). **j** Uniserial suckers of *Mastigophora brevipinnis* (Mastigophoridae, Loligosepiina), specimen MNHN 74241, La Voulte-sur-Rhone, Callovian. Scale bar: 1 mm. **k** Suckers of *Keuppia levante* (Palaeoctopodidae, Incirrata) arranged in zigzag patterns, specimen LI2011, Hâkel, Lebanon Plattenkalks, Cenomanian. Scale bar: 10 mm

Both coleoid crown groups, Octobrachia and Decabrachia, have arms equipped with suckers (Kröger et al., 2011). In contrast to the sessile suckers of octobrachians (Fig. 1h–k), the decabrachian type of sucker (Fig. 1a–d) is stalked (pedunculated) and equipped with a sclerotised cylinder (sucker ring) and a muscular piston that fits into this cylinder (e.g., Haas, 1989; Nixon, 2011). Despite this and a number of other morphological differences such as the sucker symmetry (e.g., Nixon, 2011), the two sucker types share a sucker cup (acetabulum) and an attachment ring (infundibulum). Ontogenetically, both sucker types are known to derive from transverse bulges very similar to those on the arms of *Nautilus* (Haas, 1989). These sucker primordia appear after Naef’s stage X and differentiate between stages XVIII and XX (Naef, 1921; Nolte & Fioroni, 1983). “…., *indeed, no cephalopod hatchling is known to have already transformed suckers*” (Boletzky, 2006, p. 35).

The morphology of the peg-bearing infundibulum is highly variable, particularly in the Decabrachia. The infundibulum is therefore a valuable mean for prey capture predictions (Nixon & Dilly, 1977). Kristensen (1977) mentioned “chitinous papillae” (the pegs) covering the infundibulum of *Gonatus*. However, this author did not perform a chemical analysis. Later workers found that a chitinous epithelium covers only the octopod infundibulum (Hunt & Nixon, 1981; Kier & Thompson, 2003). Nixon and Dilly (1977, p. 500) considered the outer surface of the decabrachian infundibulum to be “cuticular”, although the same authors stated that “[…] *nothing is known either of the chemical or physical properties of the infundibular cuticle, or the inner rings of the decapod sucker, except that the inner ring does not contain chitin*.” Today, as far as we know, the material lining the decabrachian infundibulum has not yet been analysed (pers. comm. Ali Miserez, March 28th, 2021). Chitins have so far been analysed only in gladiuses, beaks, and in the sucker surfaces of octopods. At least, in hematoxylin and eosin-stained sections of post-embryonal *Sepia*, both the sucker ring and the adjacent infundibulum exhibit the same colour (see Kimbara et al., 2020, Fig. 5L) suggesting a similar acidophil composition.

Up to now, nobody has seriously questioned the homology of octobrachian and decabrachian suckers. Suckers were seen initially as a synapomorphy of all coleoids, extant and extinct (Jeletzky, 1966; Naef, 1921). Later, Berthold and Engeser (1987), Haas (1989), and Young et. al. (1998) distinguished between sucker- and hook-bearing coleoids, because belemnoids (“Belemnoidea”, “Palaeocoleoidea”) were considered as sucker-less.

### Fossil record of suckers

The view that belemnoids lacked suckers was challenged by the evidence for the presence of suckers associated with Jurassic belemnoteuthids (Donovan & Crane, 1992; Fuchs et al., 2010; Mantell, 1852). The suckers of *Belemnoteuthis* and *Acanthoteuthis* are uniserial, circular, and exhibit evidence of a well-developed infundibulum (Fig. 1e–g).

Fossil suckers are either substantially preserved (phosphatised or pyritised) or imprinted in finely laminated sediments (for the fossilisation of soft parts, see: Allison, 1988; Briggs & Wilby, 1996; Fuchs, 2006a, 2006b; Clements et al., 2016; Klug et al., 2015, 2016, 2021a, 2021b; Donovan & Fuchs, 2016). Their oldest records come from La Voulte (France, early Callovian, late Middle Jurassic; Fischer & Riou, 1982a, 1982b, 2002; Kruta et al., 2016) and belong to *Mastigophora* (Fig. 1j), *Rhomboteuthis*, and *Vampyronassa*, each of which identified as gladius-bearing octobrachians (Donovan & Fuchs, 2016; Fuchs, 2014, 2020). As slightly younger specimens from Christian Malford (UK, late Callovian) confirm, *Mastigophora* possessed uniserial circular suckers without evidence of sclerotised sucker rings (Young & Vecchione, 1999; Fuchs, 2014). Other Mesozoic sucker records (in the Solnhofen and Lebanon plattenkalks) are associated with octobrachian and belemnoid remains (Fig. 1e–g, k; Fuchs & Larson, 2011a, 2011b; Fuchs et al., 2009, 2010). Cenozoic as well as Palaeozoic suckers are unknown. So far, Carboniferous localities such as the Francis Creek shale or the Bear Gulch Lagerstätte (Klug et al., 2019) yielded only arm hooks.

## Sucker rings

**Fig. 2:**
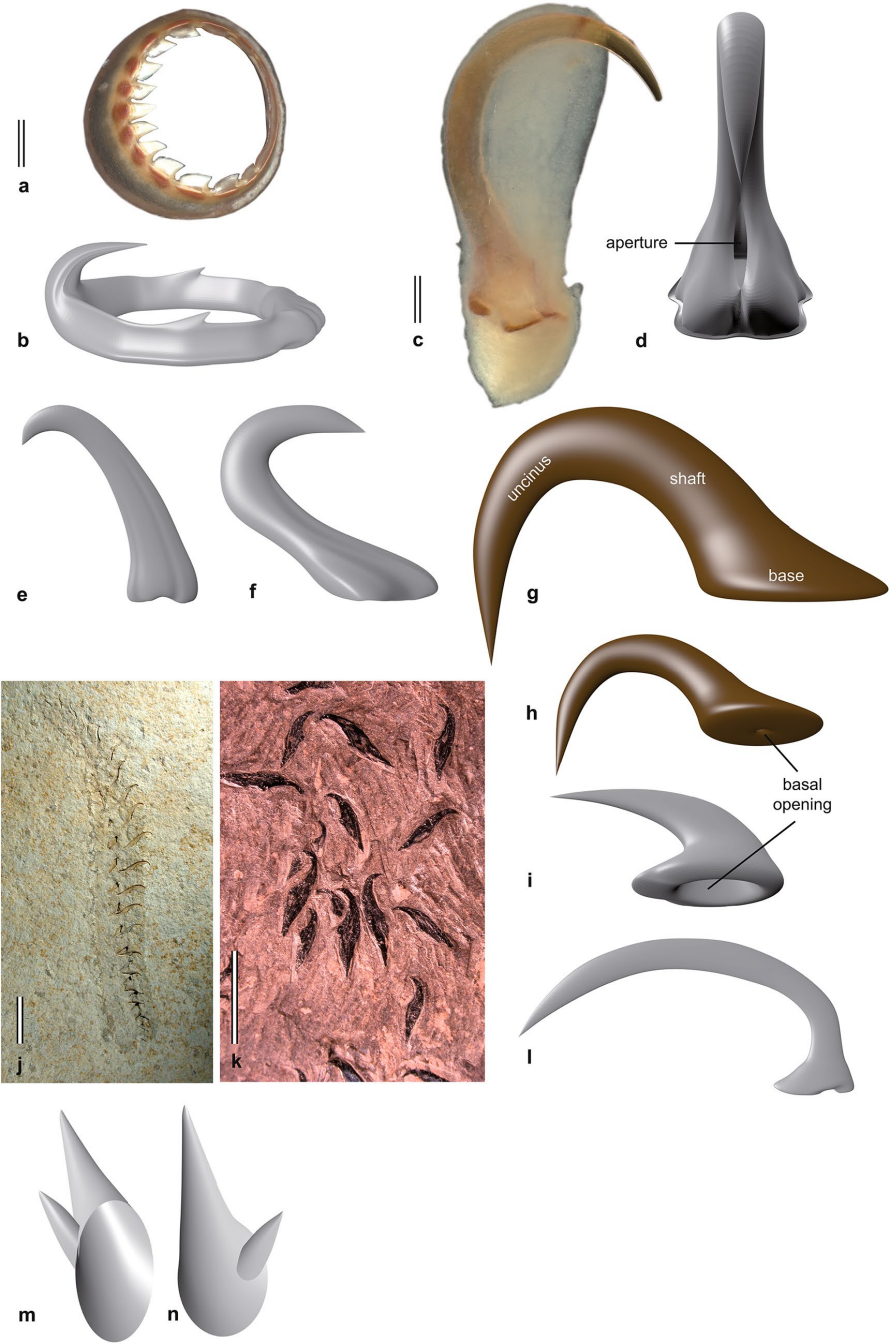
Toothed sucker rings (**a**, **b**) and arm hooks of Recent oegopsid (**c**, **d**), presumed Cenozoic oegopsid (**e**, **f**), belemnoid (**g**–**k)**, and ammonoid (**m**, **n)** cephalopods. **a** *Todarodes pacificus* (Recent, Ommastrephidae). Scale bar: 1 mm. **b** 3d reconstruction of *Mesonychoteuthis hamiltoni* (Recent, Cranchidae). **c**, **d** *Watasenia scintillans* (Recent, Enoploteuthidae), lateral (**c**) and oral (**d**) views. Scale bar: 0.2 mm. **e** 3d reconstruction of specimen NHMW1999z0050/0003 of Harzhauser (1999), Miocene, Austria. **f** 3d reconstruction of an isolated hook from the Miocene of Denmark. **g**, **h** 3d reconstruction of paragenus *Arites* (Permian–Cretaceous) in lateral (**g**) and basal (**h**) view. Note the narrow basal opening. **i** 3d reconstruction of an undetermined specimen figured in Reich (2002, Figs. 1–11) showing a comparatively wide basal opening. **j** biserially arranged hooks of *Acantoteuthis speciosa* (Belemnoteuthidae, Belemnitida), specimen MNHN B13426, Solnhofen Plattenkalks, Tithonian. Scale bar: 10 mm. **k** disarticulated hooks of *Passaloteuthis paxillosa* (Passaloteuthidae, Belemnitida), specimen SNSM26273, Schlierbach near Esslingen, Posidonia Shale, Lower Toarcian. Scale bar: 5 mm. **l** 3d reconstruction of a mega-hook (paragenus *Onychites*). **m**, **n** 3d reconstruction of *Rhaeboceras halli* (after Smith et al., 2021, Fig. 2b)

The rigid sucker rings of crown decabrachians (Fig. 2a, b) reinforce the inner surface of the acetabulum (Haas, 1989). Their oral perimeter is either smooth (e.g., Sepiolida) or may bear blunt (e.g., Sepiida) or sharply pointed teeth (e.g., Loliginida, Oegopsida).

### Ontogenetic development of sucker rings

Sucker rings appear early during ontogeny. In juvenile (post-paralarval) oegopsid *Gonatus fabricii* specimens (> 20 mm ML), the diameter of the ring aperture merely exceeds 0.01 mm (Falcon et al., 2000; Kristensen, 1977). Later stages (25 to 30 mm ML) exhibit sucker rings with an aperture of 0.12 to 0.15 mm. The apertures of adult specimens may reach between 2 and 3 mm. Though poorly documented, similar relations may be assumed for the majority of decabrachians. According to Nixon and Dilly (1977), the decabrachian (as well as the octobrachian) infundibulum grows at its periphery, i.e. from the inside outward. Haas (1989, p. 176), who focused on the sucker ring growth mode admitted that it “[…] *is not clear at the moment how the inner ring is able to increase its diameter*.” Periodical shedding of the rings similar to the infundibular lining of octopods might explain this uncertainty, but such a growth pattern is apparently unknown (Haas, 1989; pers. communication, Laure Bonnaud-Ponticelli, Vijai Dharmamony, March 2021).

### Chemical composition of sucker rings

The idea of “chitinous” sucker rings is widespread (e.g., Boletzky, 2006; Doguzhaeva & Mapes, 2014; Engeser, 1990; Engeser & Clarke, 1988; Kear et al., 1995; Naef, 1922; Nixon, 2011; Pfeffer, 1912; Stevens, 2010). Although repeatedly emphasised by various authors (e.g., Nixon & Dilly, 1977; Nolte & Fioroni, 1983; Rudall, 1955; Young et al., 1998), it is here again crucial to clarify that chitin has never been analysed in decabrachian sucker rings. Ding et. al. (2014) recently explicitly confirmed the absence of chitin in the sucker rings of the decabrachian Humboldt squid *Dosidicus gigas* (Oegopsida: Ommastrephidae).

Other authors more neutrally preferred the term “horny” sucker rings (Haas, 1989; Jeletzky, 1966; Naef, 1921, 1922; Nesis, 1987; Young et al., 1998). Accordingly, Miserez et. al. (2009) indeed determined a family of silk-like structural proteins called “suckerin” at least in the sucker rings of *Dosidicus gigas,* the bigfin reef squid *Sepioteuthis lessoniana* (Loliginida: Loliginidae)*,* and the Golden cuttlefish *Sepia esculenta* (Sepiida: Sepiidae). Apart from its well-balanced mixture of stress and strain properties, further intriguing physicochemical and thermomechanical characteristics of suckerin attracted the interests of material scientists; in particular their high elastic modulus combined with thermoplastic behaviour appears to be highly remarkable (e.g., Ding et al., 2014; Guerette et al., 2013, 2014; Hiew & Miserez, 2017).

### Fossil record of sucker rings

Except two questionable remarks (Fischer & Riou, 1982a; Mantell, 1852), sucker rings are unknown from the fossil record. Mantell (1852, Fig. 4) detected “horny” rings found in the belemnoid *Belemnoteuthis* from the Callovian of Christian Malford (UK). These circular structures have later been re-interpreted as the attachment ring (infundibulum) of belemnoid suckers as very similar imprints associated with *Acanthoteuthis* from the Solnhofen plattenkalks suggest (Fuchs et al., 2010), though a newer record appears to confirm the “ring theory” (Fig. 1e, f). Fischer and Riou (1982a) reported sucker rings visible in X-ray images of *Gramadella* from the Callovian of La Voulte. These authors therefore regarded *Gramadella* as a teuthid decabrachian, a view that is rejected herein (see above). Instead, *Gramadella* is more likely a gladius-bearing octobrachian similar to *Proteroctopus* that is evidently missing sucker rings (Kruta et al., 2016). The general absence of sucker rings in Mesozoic gladius-bearing octobrachians have repeatedly been pointed out (Donovan & Fuchs, 2016; Engeser & Clarke, 1988; Jeletzky, 1966; Naef, 1922). The ring-like structures in the centre of suckers appear flat rather than cylindrical and might alternatively been interpreted as the (chitinous?) infundibulum of an octobrachian (Fuchs, 2020).

## Oegopsid arm hooks

The arms and/or tentacles of some oegopsid families (Enoploteuthidae, Onychoteuthidae, Lepidoteuthidae, Octopoteuthidae, Gonatidae, Cranchidae) are equipped with claw-like structures (Fig. 2c, d). These hooks are covered on the outside by a thin translucent dermal sheath; only the tips of the claws are free. Shape, number, and arrangement of these commonly called hooks vary considerably even along arms and tentacles (Nixon, 2011). The claw characteristically curves towards the hook base, which still exhibits an aperture as a reminiscence of the former sucker ring. Normally, the shape of oegopsid hooks is not subject to sexual dimorphism; however, it is worth mentioning that only the males of *Lepidoteuthis grimaldii* (Oegopsida: Lepidoteuthidae) possess a single, significantly enlarged, sabre-like hook on each of their dorsolateral arms (Jackson & O´Shea, 2003). Both morphological as well as molecular phylogenies suggest that hook-bearing oegopsids do not form a monophyletic grouping, which implies multiple independent claw-like outgrowths of sucker ring teeth.

### Development of oegopsid hooks

Although nineteenth century workers such as Owen (1844, p. 81) were already aware of the fact that hooks develop from sucker rings, the knowledge about the ontogenetic development of hooks is very scanty (Appelöff, 1893; Naef, 1921, p. 129–133, pl. 12). All we know about the morphogenesis of oegopsid hooks comes from a study by Kristensen (1977), wherein the armature of different growth stages (mantle lengths) of *Gonatus fabricii* have been compared and tracked. Despite some problematic interpretations, he ascertained that the hook is a derivate of the ring wall rather than from the infundibulum (= Kristensen’s attachment ring). The same author also remarked on page 113 that “*Transitional stages […] were impossible to find, so the development between the two stages is probably very rapid*.”

It is also worth citing Pfeffer (1912, p. XVII), who presumed that “*Despite a certain similarity in form and development in various divisions *(*onychoteuthids*, *enoploteuthids*, *gonatids*, *cranchiids*), *profound differences in hook formation can be found*” [translated from German]. Pfeffer (1912) announced a detailed explanation of these differences in hook formation, which was never published.

### Chemical composition of oegopsid hooks

Surprisingly, the chemistry of oegopsid hooks have not been analysed yet. Since they are modifications of sucker rings, it is reasonable to assume that hooks are made of suckerin as well. A chitinous composition as occasionally presumed (e.g., Young & Harman, 1998) appears questionable.

### Fossil records of oegopsid hooks

Fossil hooks that show unambiguous characteristics of oegopsid hooks such as the reminiscence of the ring aperture are unknown. However, there are at least four Cenozoic specimens that have tentatively been interpreted to belong to oegopsid squids (Fuchs & Hoffmann, 2017). Harzhauser (1999) found two fragmentary specimens in the residues of middle Miocene (Langhian) sediments from Austria (Fig. 2e). Unlike modern oegopsid hooks, the tip of this fossil hook from the Paratethys does not curve towards the hook base, but away from it.

Two additional specimens recently discovered in middle Miocene deposits from Denmark are currently under investigations (personal observations in cooperation with Mette Stemann and Jan Rasmussen, Copenhagen). They differ from the Paratethys specimens particularly in having a tip that is curved towards the base, i.e. forming an uncinus that is typical for oegopsid hooks (Fig. 2f). In both Miocene hook types, evidence of a basal opening or a ring aperture is missing.

## Belemnoid arm hooks

Hooks superficially similar to oegopsid hooks also furnished the oral surfaces of the arms of belemnoid coleoids (e.g., Engeser & Clarke, 1988; Fuchs & Hoffmann, 2017; Klug et al., 2010, 2016, 2020, 2021b). Although the detailed terminology of belemnoid and oegopsid hooks is different (owing to morphological and developmental differences), both hook types generally share a base, a shaft, and the uncinus (Fig. 2g–l). Also similar to oegopsid hooks, it is assumed that belemnoid hooks were covered from outside by a thin dermal sheath (Engeser, 1987; Fuchs & Hoffmann, 2017). This assumption is based on the presence of a so-called orbicular scar, which is commonly considered to represent the border between covered and uncovered parts of the uncinus.

We informally distinguish between micro- (< 5 mm) and mega-hooks (> 5 mm). Micro-hooks are abundant, variable in shape, biserially (rarely uniserially, e.g., *Chondroteuthis*) arranged, present on each of the ten arms, and evidenced for at least members of the Phragmoteuthida and Belemnitida (assumed for Aulacoceratida and Diplobelida). Mega-hooks (Fig. 2l) are clasp-like, less abundant, less diverse, and autapomorphic for the Belemnitida (e.g., Fuchs, 2006a; Klug et al., 2021b; Reitner & Urlichs, 1983; Riegraf et al., 1984). The detailed position within the arm crown as well as the function of this certainly specialised hook pair is still debated (Fuchs, 2006a; Fuchs & Hoffmann, 2017; Klug et al., 2021b). Isolated hooks from localities without any evidence of belemnoid body fossils are classified in a parataxonomic system (Kulicki & Szaniawski, 1972).

In contrast to oegopsid hooks, the uncini of belemnoid hooks are usually curved away from the base, which never exhibits an aperture in its centre. Another typical feature of belemnoid hooks is a cavity in the shaft that terminates in the basal opening. The basal opening of this cavity may be small, hole-like or wide, ring-like (Reich, 2002; Fig. 2h, i). The oldest belemnoid hooks (*Jeletzkya*) come from the Carboniferous Francis Creek shale; the last unambiguous belemnoid hooks occur in Maastrichtian deposits (Fuchs & Hoffmann, 2017).

### Ontogenetic development of belemnoid hooks

Boletzky (1999, p. 8) remarked that the formation of belemnoid hooks represents a “*paleomorphological problem*”. Indeed, the formation of belemnoid hooks is still unknown. Since belemnoid sucker rings are unknown (see above), many authors agreed that belemnoid hooks did not develop through rings (e.g., Engeser & Clarke, 1988; Young et al., 1998). However, fragments of hooks broken above the base or the lower shaft appear distinctly ring-like. Periodic shedding of hooks cannot be excluded since the bulk of fossil hooks has been found isolated.

### Chemical composition of belemnoid hooks

Belemnoid hooks are either preserved as empty imprints or, if substantially conserved, often carbonised (e.g., Doguzhaeva et al., 2007; Fuchs & Hoffmann, 2017; Klug et al., 2010). Their original composition is still uncertain mainly owing to diagenetic effects (e.g., recrystallisation, impregnation, carbonisation). Engeser and Clarke (1988) assumed a chemical difference between decabrachian sucker rings and belemnoid hooks, whereas Haas (1989) considered no difference. For a long time, the unconfirmed assumption of “chitinous” sucker rings (see above) has also been propagated in the palaeontological literature (e.g., Berthold & Engeser, 1987; Doguzhaeva & Mapes, 2014; Engeser & Clarke, 1988; Hoffmann & Stevens, 2020; Klug et al., 2010). Engeser and Clarke (1988) argued—though based on the erroneous idea of chitinous oegopsid hooks—that belemnoid hooks lacked chitin. This view is rooted in their observation that, during the Mesozoic, hooks are more abundant in predator stomachs than chitinous beaks, while this ratio is inverse today (Engeser & Clarke, 1988). Based on their EDX analyses, Doguzhaeva et. al. (2007) suggested an involvement of chitin despite chemical differences between gladius, beaks and hooks from the same locality. These authors probably referred to variation of the protein–chitin ratio known to occur in many cephalopod tissues (Hunt & Nixon, 1981). In this context, it is essential to note that in the Nusplingen Plattenkalks (Late Jurassic, SW Germany), belemnoid hooks are exclusively known as imprints (external and internal moulds) although chitinous structures such as gladiuses and beaks are well preserved (Klug et al., 2010). Similarly, Late Cretaceous deposits in Hokkaido (northern Japan) are known to contain well-preserved cephalopod beaks (Tanabe, 2012), while hooks are unknown—despite the (admittedly rare) presence of belemnoids (*Longibelus*, *Conoteuthis*).

Other authors alternatively assumed belemnoid hooks to be “horny” (e.g., Haas, 1989; Kulicki & Szaniawski, 1972). The use of this somewhat vernacular term refers to the absence of chitin and the sole presence of structural proteins (scleroproteins). Participation of conchiolin as assumed by Kulicki and Szaniawski (1972) appears unlikely. Conchiolin is a typical constituent of the mollusc shell, specifically of the outermost (periostracal) layer. Stevens (2010) discussed cartilage as a possible material for belemnoid hooks, but, apart from inadequate mechanical properties of cartilage, the preservational potential of cartilaginous arm hooks would be strongly reduced. For instance, cephalic cartilages are preserved only occasionally and exclusively in very few localities (e.g., Solnhofen and Lebanon plattenkalks; Fuchs & Larson, 2011a, 2011b; Klug et al., 2016). Vice versa, cephalic cartilages are very rare in localities with well-preserved arm hooks (e.g., Posidonia Shale).

## Ammonite arm hooks

Hook-like structures have been described repeatedly from Late Cretaceous ammonites (Kennedy et al., 2002; Kruta et al., 2013, 2020; Landman & Waage, 1993). These structures were found in body chambers of the scaphitids *Hoploscaphites* and *Rhaeboceras* from the Campanian and Maastrichtian of the US-American Western Interior (Kruta et al., 2020; Smith et al., 2021). Based on X-ray studies, Smith et. al. (2021) confirmed the existence of five morphotypes, which are generally typified by unicuspidate or bicuspidate uncini (Fig. 2m, n). The arrangement, chirality, paired occurrences and presence in many specimens is good evidence that these structures indeed belong to the ammonites. Their linear arrangement and similarity to belemnoid and oegopsid arm hooks suggest that either tentacles or reproductive organs of one sex were equipped with these hooks (Kruta et al., 2020). Despite detailed differences to belemnoid hooks, they share the hollow base, the small size relative to the whole animal, and the paired appearance.

## Cirri

**Fig. 3:**
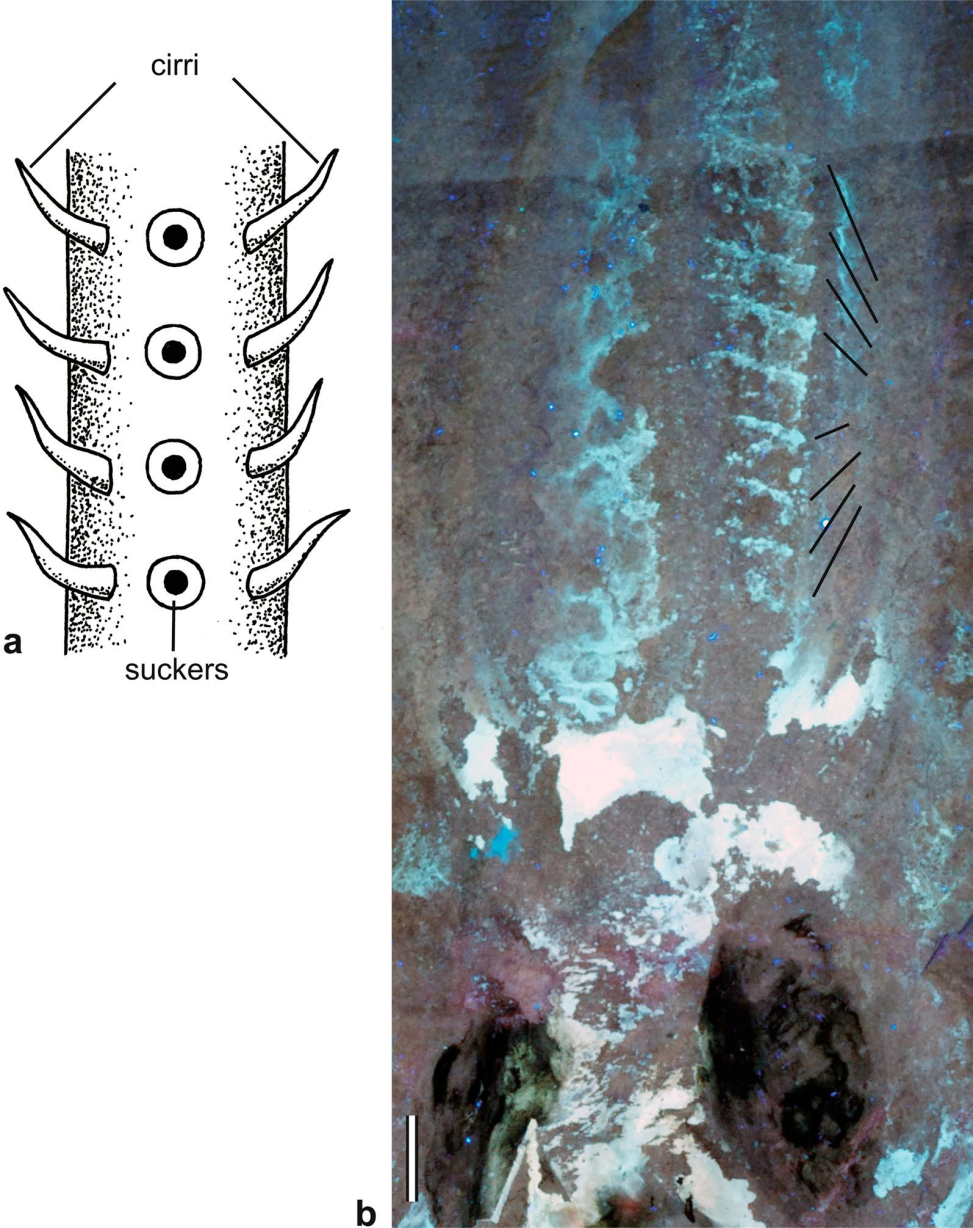
Cirri of vampyromorph and cirrate octopods. **a** Schematic drawing illustrating the biserial arrangement of cirri. **b** Fossilised cirri along the arms of *Glyphiteuthis* sp. (Trachyteuthidae, Teudopseina), specimen BHI 5813, Hâdjoula, Cenomanian. Scale bar: 10 mm

Strand- or filament-like cirri (Fig. 3a, b) are not or at least poorly sclerotised paired appendages on the arms of modern and extinct octobrachians (e.g., Fuchs, 2006a; Hoving & Robison, 2012; Klug et al., 2015). The paired arrangement of these tactile organs on the flanks of a single row of suckers and the proportions relative to the arm raise the question for their possible homology. Haas (1989), Young et. al. (1998), and Fuchs et. al. (2013a) homologised them with belemnoid hooks, while Boletzky (2006, p. 35) stated: “*Indeed, vampyromorph (and cirroctopod) cirri are unlikely homologues of belemnoid hooks*”.

Fossilised cirri have been recorded in association with Mesozoic octobrachians (*Plesioteuthis* from the Late Jurassic Solnhofen Plattenkalks and *Glyphiteuthis* from the Late Cretaceous Lebanon Plattenkalks; Bandel & Leich, 1986; Fuchs & Larson, 2011a, 2011b; Klug et al., 2015; Donovan & Fuchs, 2016). In a *Plesioteuthis* from the Kimmeridgian of Painten, the dark organic preservation of cirri suggests a light (rudimentary?) sclerotisation (cf. Klug et al., 2015) or a unique preservation of dermal pigments.

## A posteriori: character state reconstructions

In contrast to octobrachian and decabrachian suckers, whose homologous nature was never questioned seriously, the occurrence and homologue nature of arm hooks has created some confusion in the literature. This confusion has its roots deep in pioneering coleoid research. Münster (1837), for instance, determined Jurassic *Onychoteuthis* with hooks without providing evidence of hooks. A brief historical review about the (non)homology of cephalopod hooks is given in Table 1.

**Table 1:** Scenarios on the (non)homology of cephalopod hooks, cirri, and suckers and their supporters (the role of ammonoid hooks not included)

	Scenario 1	Scenario 2
Belemnoid hooks vs. suckers	Belemnoid hooks derived from primitive suckers	Belemnoid hooks evolved de novo
	→ Belemnoid hooks and suckers are **homologous**	→ Belemnoid hooks and suckers are **convergent**
	Naef (1922, p. 165, 188), Jeletzky (1966, p. 138), Haas (1989, p. 182), Boletzky (1999, p. 8, 2006: p. 35), herein	Berthold and Engeser (1987, p. 198), Engeser and Clarke (1988, p. 142), Young et al. (1998), Fuchs et. al. (2010, 2013b)

	Scenario 1A	Scenario 1B	
Belemnoid hooks vs. primitive sucker rings	Belemnoid hooks derived from primitive sucker rings	Belemnoid hooks evolved from suckers without sucker rings	
	→ Belemnoid hooks and sucker rings are **homologous**	→ Belemnoid hooks and sucker rings are **convergent**	
	Naef (1922, p. 188), Haas (1989, p. 182–183), herein	Naef (1922), Jeletzky, 1966), Berthold and Engeser (1987, p. 198), Young et al., (1998, p. 395), Fuchs et. al. (2010, 2013b)	
Belemnoid hooks vs*.* decabrachian sucker rings	Decabrachian sucker rings adopted from belemnoid sucker rings	Decabrachian sucker rings evolved de novo	
	→ Belemnoid and decabrachian sucker rings are **homologous**	→ Belemnoid and decabrachian sucker rings are **convergent**	
	Herein	Berthold and Engeser (1987, p. 198), Engeser and Clarke (1988, p. 142), Young et al. (1998), Fuchs et. al. (2010, 2013b)	
Belemnoid hooks vs oegopsid hooks	Oegopsid hooks derived from toothed sucker rings		
	→ Belemnoid and oegopsid hooks are **parallel** developments	→ Belemnoid and oegopsid hooks are **convergent**	
	Naef (1922), herein	Berthold and Engeser (1987, p. 198), Engeser and Clarke (1988, p. 142), Young et al. (1998), Fuchs et. al. (2010, 2013b)	
Belemnoid hooks vs. octobrachian cirri	Octobrachian cirri derived de novo	Octobrachian cirri derived from belemnoid hooks	
	→ Belemnoid hooks and octobrachian cirri are **convergent**	→ Belemnoid hooks and octobrachian cirri are homologous	
	Boletzky (2006), herein	Berthold and Engeser (1987, p. 198), Young et al., (1998, p. 395), Fuchs et. al. (2010, 2013b)	

### Nineteenth century view

Voltz (1830) like many nineteenth century workers (e.g., Münster 1839; Orbigny 1845, Woodward, 1851) classified living decabrachians along with belemnitids. These workers therefore implicitly regarded belemnoid and oegopsid hooks as homologues.

### Twentieth century view

Though Naef (1922) was a follower of Voltz’ “Belemnitid root stock” theory, he stated on page 187: “*As far as arms are sufficiently well preserved in the fossil record of belemnoids, …,these arms bear structures resembling the hooks of recent teuthoids; they can only be interpreted in analogy to the latter*.” Jeletzky (1966, p. 138), also a follower of Voltz (1830), agreed: “[*…*] *the transformation of one part of the suckers into arm hooks occurred several times in not directly related major Coleoidea taxa*.”

Donovan (1977) was one of the first to consider belemnitids as an extinct group without descendants. He suggested that crown decabrachians (as well as octobrachians) derived from phragmoteuthid belemnoids (see also Berthold & Engeser, 1987; Doyle et al., 1994). Such a topology made it easier to reject a homology between belemnitid and oegopsid hooks. In their acknowledged review on cephalopod arm hooks, Engeser and Clarke (1988, p. 146) stated: *“There is no doubt that the hooks have evolved more than once and the extinct precursors of the recent hooks are not known to us*.” Additional support for a convergent evolution came from Young and Harman (1998) and Young et. al. (1998), who assumed multiple independent hook developments even within the Oegopsida.

Haas (1989, p. 184), by contrast, summarised in his studies on comparative morphology and anatomy of suckers as follows: “[…] *one can trace a basic set of common features. The basic set of homologous characters is derived from embryology and from “constructive simplification”* […] *and can be seen in a stage consisting of transverse bulges on the ventral side of the arms similar to* Nautilus *but provided with some sort of suction chamber.* […] *The further evolution may have led* […] *to rather different types of suckers in the two living coleoid taxa. The similarities between the arm hooks of the Belemnoidea and the horny rings of the Decabrachia are due to parallelism.*”.

### Twenty-first century view

The revival of the “belemnitid root stock theory” of Voltz (1830) was initiated by Hewitt and Jagt (1999) who first suggested a separate origin of Sepiida and Spirulida within diplobelid belemnoids (based on the assumption of separate development of a caecum; see Fuchs, 2019). Later, this view received support from several phylogenetic analyses (Fuchs, 2019; Fuchs & Iba, 2015; Fuchs, et al., 2013b, 2016; Klug et al., 2016; Kröger et al., 2011; Sutton et al., 2015). Although this actual topology of the Coleoidea theoretically enables a direct derivation of oegopsid from belemnitid/diplobelid hooks, the idea of multiple developments of arm hooks is still widely accepted (see Fuchs & Hoffmann, 2017).

The idea of a sister group relationship between Ammonoidea and Coleoidea is older, but regarding ammonoids as stem coleoids is a propagated view of the last decade (Fuchs, 2019; Kröger et al., 2011), in which it is necessary to include ammonoid hooks when considering the homology of cephalopod arm hooks.

## Discussion

A priori character state reconstructions of the cephalopod arm armature are hampered by a patchy fossil record and thus ambiguous. We therefore focus in the following on a posteriori character state reconstructions that are based on the phylogeny of the Coleoidea as, e.g., suggested by Kröger et. al. (2011).

A sister group relationship between “Belemnoidea” (including among others Belemnitida and Diplobelida) and the Neocoleoidea is no more tenable, because the autapomorphies previously used to establish both taxa are questionable (e.g., presence of suckers). The taxon Neocoleoidea is monophyletic only after the inclusion of proostracum-bearing belemnoids (Phragmoteuthida, Belemnitida, Diplobelida) with Phragmoteuthida containing the last common ancestors of the Decabrachia and Octobrachia (e.g., Doyle et al., 1994). A whole set of arguments speaks for this inclusion: (1) arm modification (Boletzky, 1992, 1999); (2) proostracum (Fuchs & Iba, 2015; Fuchs et al., 2010, 2013b, 2016); (3) caecum (Fuchs, 2019), and last but not least (4) evidence of belemnitid suckers (Fuchs et al., 2010). When including these into cladistic analyses (e.g., Kröger et al., 2011; Sutton et al., 2015), this hypothesis finds sufficient support. Also, it is worth noting that there are no reasons to assume multiple origins of belemnoid hooks (by contrast to oegopsid hooks). As another consequence of paraphyletic Belemnoidea, it is coherent to consider belemnoid taxa with a tubular final chamber (Aulacoceratida, Donovaniconida, and Hematitida) as stem neocoleoids and the ectocochleate Bactritoidea and Ammonoidea as stem coleoids.

### Evolutionary development of the coleoid armature

Independently from the phylogeny used (i.e. Neocoleoidea with or without proostracum-bearing belemnoids), the phylogenetic bracket generally opens the possibility that primitive sucker cups evolved in the coleoid stem lineage. If suckers evolved later in the stem lineage of the Neocoleoidea, a morphogenetic program for suckers did not exist in stem coleoids (Ammonoidea and Bactritoidea). Carboniferous Donovaniconida and Hematitida might have lacked suckers as well. Primitive suckers then possibly evolved in early aulacoceratids (Permian to Jurassic) or phragmoteuthids (?Permian, Triassic to Jurassic). Such a late development would challenge the idea whereupon suckers derived from transverse bulges present in the nautiloid lineage (see Haas, 1989). While Haas (1989) and Boletzky (1999) assumed an early evolution of suckers, authors like Berthold and Engeser (1987) and Young et. al. (1998) discussed a later evolution in the stem lineage of neocoleoids. We here follow Haas (1989) and Boletzky (1999), who considered the possibility that ammonoid and bactritoid arms were equipped with suckers.

**Fig. 4:**
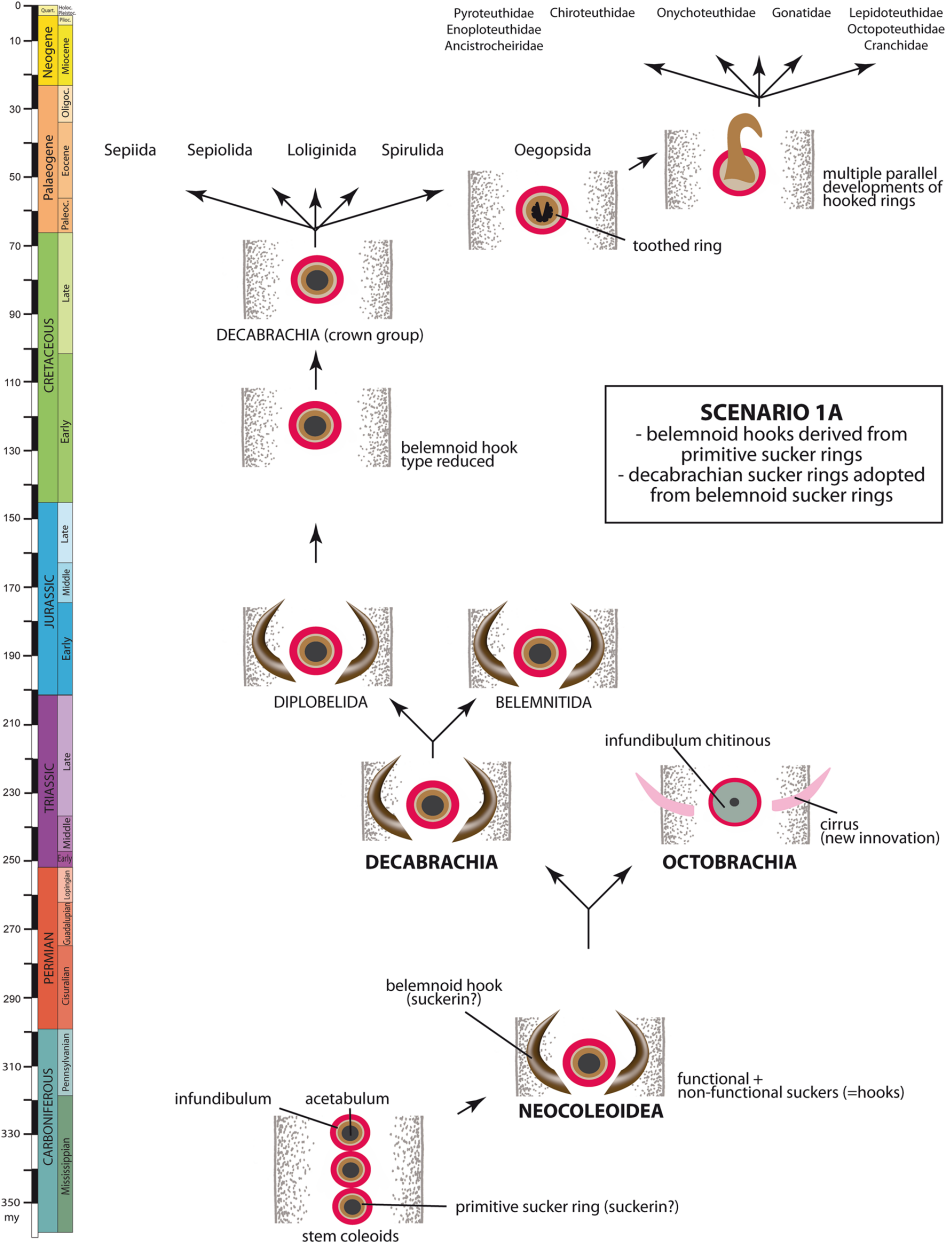
Evolutionary development of the cephalopod arm armature: scenario 1A

**Fig. 5:**
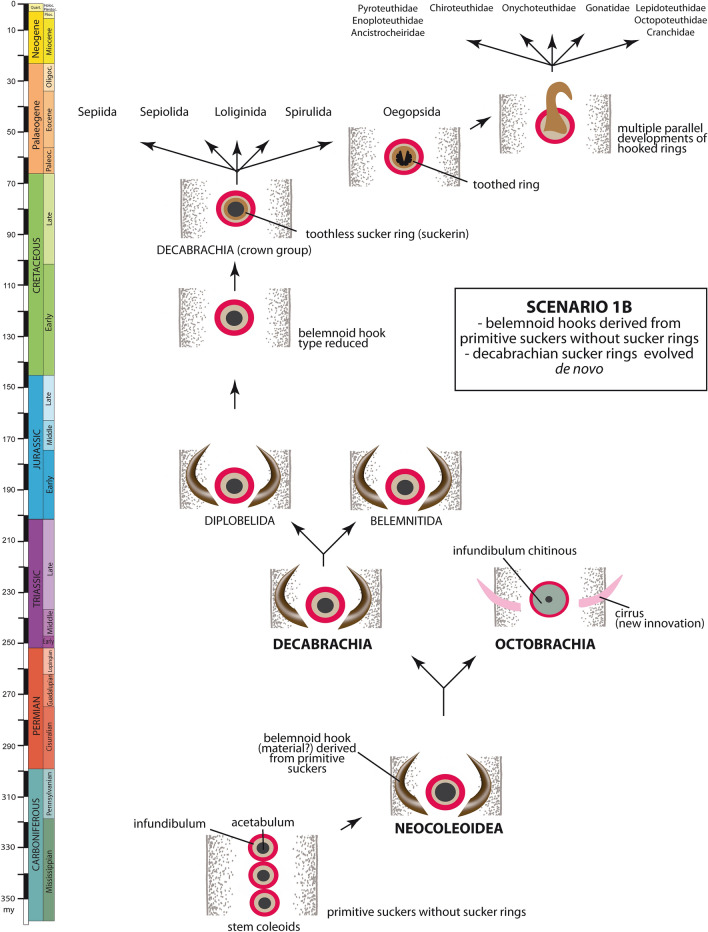
Evolutionary development of the cephalopod arm armature: scenario 1B

**Fig. 6:**
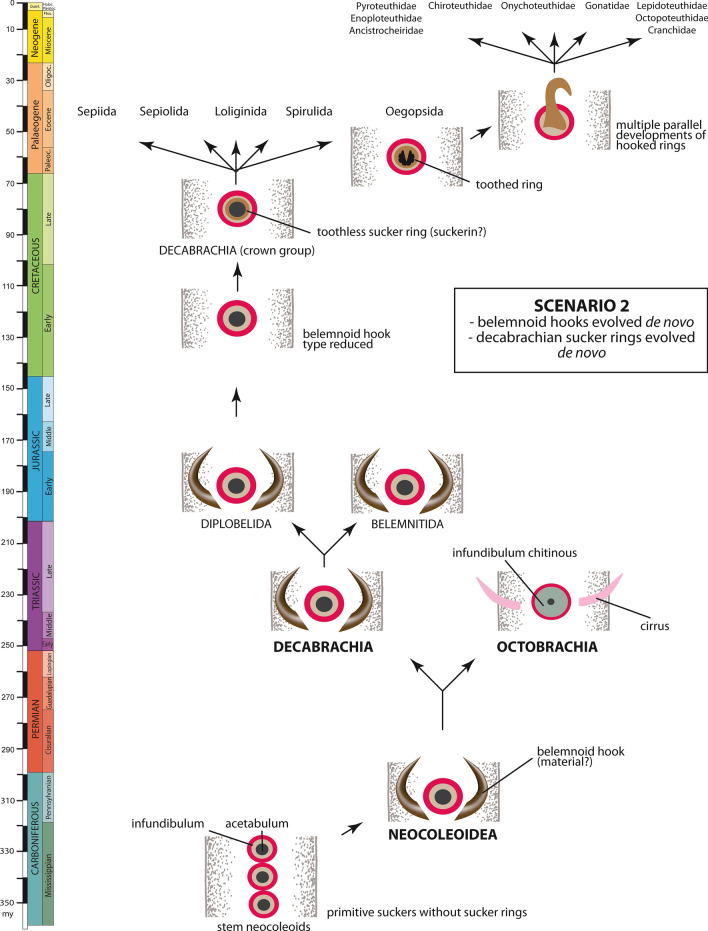
Evolutionary development of the cephalopod arm armature: scenario 2

Regarding belemnitid and diplobelid belemnoids as stem decabrachians as well as evidence of ammonoid arm hooks generally impacts the evolutionary development of the belemnoid type of arm hook (see discussion of hooks in scaphitids; Kruta et al., 2020). The following scenarios are conceivable (Table 1, Figs. 4, 5, 6):

### Scenario 1: belemnoid hooks derived from primitive suckers

This scenario suggesting that belemnoid hooks and suckers are homologous was assumed by Naef (1922, p. 165, 188), Jeletzky (1966, p. 138), Haas (1989, p. 182), and Boletzky (1999, p. 8, 2006, p. 35). This means that belemnoids (or at least belemnitids) possessed both functional suckers and modified, non-functional suckers (= hooks). The evolution of the belemnoid type of hook from primitive suckers may have occurred in two ways:

### Scenario 1A: belemnoid hooks derived from primitive (toothless) sucker rings (Fig. 4)

Cross sections through the proximal part of the shaft of belemnoid hooks suggest that belemnoid hooks grow through a ring stage. Already Naef (1922, p. 188) assumed the basal opening of belemnoid hooks as a rudimentary sucker ring. Apart from this mutuality, Haas (1989, p. 180) emphasised the microstructural similarity between belemnoid hooks and decabrachian sucker rings Hence, the rejection of a hypothetical transformation from a ring to a hollow hook by Engeser & Clarke (1988, p. 135) appears premature.

The derivation of decabrachian sucker rings from the belemnoid armature is conceivable. Either crown decabrachians may have simply adopted the sucker rings from their belemnoid (?diplobelid) ancestors or the decabrachian type of sucker ring represents a rudimentary belemnoid hook. A hypothetical transformation from an abnormal (rudimentary) belemnoid hook type to the decabrachian type of sucker ring is based on Late Cretaceous belemnoid hooks typified by a wide basal opening (Fig. 2i).

Anyway, regarding belemnoid hooks and decabrachian sucker rings as homologues implies that belemnoid hooks were likewise made of suckerin. The main component of the decabrachian sucker rings would have then originated along with the first appearance of belemnoid hooks during the Carboniferous in stem neocoleoids at the latest.

In this scenario, oegopsid and belemnoid hooks automatically represent parallel developments (= independent evolution of homologous characters) as indirectly advocated by Naef (1922). If ammonoid hooks were likewise derivates of sucker rings, all hook types were made of suckerin and arose from parallel evolution. Such an assumption would imply that sucker rings evolved during the earliest Devonian as the putative divergence of bactritoids and ammonoids suggests. Consequently, the innovation of functional, still ring-less suckers would have happened earlier. Molecular clock analyses that suggest a split between the nautiloid and coleoid lineage during the Silurian corroborate the option of a very early evolution of the coleoid type of sucker (Tanner et al., 2017; López-Córdova et al., 2021).

### Scenario 1B: belemnoid hooks did not derive from sucker rings (Fig. 5)

If belemnoid hooks evolved from primitive suckers, but independently from sucker rings, they must have arisen from an unknown sucker structure. It is possible that belemnoid hooks represent outgrowths of the infundibulum by contrast to sucker rings, which develop from the inner (acetabular) sucker wall. Belemnoid hooks were consequently composed of a material other than suckerin or chitin.

Regarding the sucker rings as an autapomorphy of crown decabrachians implies that the suckers of Maastrichtian sepiids (*Ceratisepia*), as the oldest unambiguous evidence of crown decabrachians, were reinforced with rigid rings. Such a comparatively late origin of sucker rings suggests that they evolved in a hook-less belemnoid subgroup (Diplobelida?, see Hewitt & Jagt, 1999; Fuchs, 2019; Fuchs et al., 2013b) at a time when the diversity and abundance of hook-bearing belemnitids and diplobelids already decreased. Belemnoid and oegopsid hooks were consequently the result of a convergent evolution (rather than parallel evolution as in scenario 1A) as considered by Berthold and Engeser (1987), Engeser & Clarke, 1988), Young et. al. (1998), and Fuchs (2010); Fuchs et al., (2013a). With regard to ammonoids, their type of arm hooks derived in this scenario from an uncertain structure, whose morphogenetic origin and chemical composition remains dubious.

### Scenario 2: belemnoid hooks did not derive from suckers (Fig. 6)

Scenario 2 is identical to the evolutionary steps reconstructed in scenario 1B except that belemnoid hooks developed independently from the sucker complex. The majority of authors argued for such a relationship (Berthold & Engeser, 1987; Engeser & Clarke, 1988; Fuchs 2010, Fuchs et al., 2013a; Young et al., 1998). If belemnoid hooks are dealt with as a new arm armature of Palaeozoic stem neocoleoids (Hematitida, Donovaniconida, Aulacoceratida), decabrachian sucker rings represent a novelty as well. Also, one might interpret octobrachian cirri as derivates of belemnoid hooks. The belemnoid hook type must have been reduced in at least one group of belemnitids or diplobelids that gave rise to crown decabrachians. Belemnoid and oegopsid hooks are accordingly the result of convergent evolution.

## Conclusions and future perspectives

We consider scenario 2 (belemnoid hooks did not derive from suckers, i.e. they evolved de novo and become reduced without related structures) as less likely, whereas scenario 1 (belemnoid hooks derived from primitive suckers) is—contrary to the current opinion and in agreement with Naef (1922), Jeletzky (1966), Haas (1989), and Boletzky (1999, 2003, 2006)—better supported. Belemnoid hooks are accordingly homologous to suckers rather than to cirri. In detail, we tend to support scenario 1A, which implies that belemnoid hooks derived from sucker rings. Belemnoid hooks accordingly evolved through primitive toothless sucker rings. Subsequently, crown decabrachians either directly inherited the smooth sucker rings or adopted ring-shaped rudiments of belemnoid hooks. This latter idea is mainly based on the wide basal opening observed in Late Cretaceous belemnoid hooks. Hence, belemnoids possessed both modified and non-modified suckers. Oegopsid and belemnoid hooks thus resulted from parallel evolution, a frequently observed phenomenon in cephalopods. An early evolution of sucker rings would also easily explain the occurrence of hooks along ammonoid arms. Admittedly, the virtual lack of sucker rings in the fossil record represents the weak point in this scenario, but the limited preservation of sucker rings is obvious. The Cenozoic fossil record has not delivered sucker rings yet, although rigid and well-sclerotinised sucker rings must have been existed since the Maastrichtian at least.

### Ring/hook growth modus

Our conclusion may be verified by new insights about the growth of decabrachian rings and hooks, which is not yet fully understood. The absence of transitional stages between a typical multi-toothed sucker ring and a hook morphologically close to the adult hook as well as the assumption of a very rapid development might alternatively point to periodical shedding and the formation of new larger rings and hooks—similar to the new formation of the octopod infundibulum lining. However, this is apparently not the case. As a result of this, the following questions about the mode of sucker ring growth are still open:

1. How can juvenile sucker rings with a tiny diameter develop into a ring with a larger diameter? How is it possible to increase the diameter of a stiff sclerotised structure? Stretching of the ring wall might be one option, but the ring wall is said to grow only in thickness (from the inside outward) and not by adding intermediate material. Resorption of the inner ring wall might be another option to increase the diameter, but such a pattern would afford an appropriate epithelium, which apparently does not exist.
2. What happened to the remaining teeth? Are they resorbed or covered with additional material?

Similar questions concern the formation of belemnoid hooks. Two difficult questions are crucial for the assessment of belemnoid hooks:

1. What was the original composition of belemnoid hooks?
2. Is carbonisation more likely in purely proteinous or chitinous structures?

Both questions concern the general mode of formation: each of the structures under discussion probably share the absence of continuous growth (no transitional stages, no steady transformation).

### Chemistry of belemnoid and ammonoid hooks

The lack of substantially preserved belemnoid arm hooks in localities (e.g., Late Jurassic of Nusplingen, Late Cretaceous of Hokkaido) with a demonstrably good preservation of originally chitinous structures as well as the fact that oegopsid hooks do not contain chitin (see above) let a chitinous composition of belemnoid arm hooks appear improbable. Instead, we are inclined to consider an interplay of structural proteins such as suckerin as it is the case in oegopsid hooks.

## Data Availability

Does not apply.
